# Effect of Octacalcium Phosphate Crystals on the Osteogenic Differentiation of Tendon Stem/Progenitor Cells In Vitro

**DOI:** 10.3390/ijms24021235

**Published:** 2023-01-08

**Authors:** Xianchen Liu, Yukari Shiwaku, Ryo Hamai, Kaori Tsuchiya, Tetsu Takahashi, Osamu Suzuki

**Affiliations:** 1 Division of Craniofacial Function Engineering, Tohoku University Graduate School of Dentistry, Sendai 980-8575, Japan; 2 Division of Oral and Maxillofacial Reconstructive Surgery, Tohoku University Graduate School of Dentistry, Sendai 980-8575, Japan

**Keywords:** octacalcium phosphate, Ca-deficient hydroxyapatite, tendon stem/progenitor cells, tendon–bone repair

## Abstract

Synthetic octacalcium phosphate (OCP) activates bone tissue-related cells, such as osteoblasts, osteoclasts, and vascular endothelial cells. However, the effect of OCP on tendon-related cell activation remains unknown. This study examined the response of rat tendon stem/progenitor cells (TSPCs) to OCP and related calcium phosphate crystals in vitro. TSPCs were cultured with OCP and Ca-deficient hydroxyapatite (CDHA) obtained from the original OCP hydrolysis to assess the activity of alkaline phosphatase (ALP) and the expression of osteogenesis-related genes. Compared with CDHA, the effect of OCP on promoting the osteogenic differentiation of TSPCs was apparent: the ALP activity and mRNA expression of *RUNX2*, *Col1a1*, *OCN*, and *OPN* were higher in OCP than in CDHA. To estimate the changes in the chemical environment caused by OCP and CDHA, we measured the calcium ion (Ca^2+^) and inorganic phosphate (Pi) ion concentrations and pH values of the TSPCs medium. The results suggest that the difference in the osteogenic differentiation of the TSPCs is related to the ionic environment induced by OCP and CDHA, which could be related to the progress of OCP hydrolysis into CDHA. These results support the previous in vivo observation that OCP has the healing function of rabbit rotator cuff tendon in vivo.

## 1. Introduction

The structure of octacalcium phosphate (OCP) is composed of alternating apatite layers stacked with hydrated layers [1]. OCP is a secondary precursor to hydroxyapatite (HA) after the initial formation of amorphous calcium phosphate (ACP) from supersaturated solutions [2]. OCP has been advocated as a precursor for bone and tooth apatite crystals [3,4,5,6]. Although the structure of OCP is closely related to that of HA [1], the properties of OCP as a bone substitute are different from those of HA [7]. The implantation of OCP granules enhances intramembranous bone formation more than that of HA granules, even if the bone defect size is larger than the critical defect size, which does not heal spontaneously [8,9,10]. Comparative studies of OCP and HA have found that OCP promotes bone regeneration, while OCP undergoes phase conversion to Ca-deficient HA (CDHA) under physiological conditions [6,8,11].

OCP enhances the osteoblastic differentiation of bone marrow cells [8,12,13]. OCP enhances the osteoblastic cell differentiation of mouse bone marrow stromal cells in vitro [14] and bone formation in rat calvaria defects [15] in a dose-dependent manner. Furthermore, OCP can enhance mesenchymal stem cell (MSC) differentiation into osteoblasts [16] and osteocytes [17], migrate macrophages [18], form osteoclasts from bone marrow macrophages with osteoblasts [19], and form capillary-like tubes by human umbilical vein endothelial cells (HUVECs) [20]. The structural and chemical analyses of OCP and the supernatants after incubation suggest that OCP provides an inorganic ion chemical environment around the OCP that is appropriate for activating these cellular activities [11,14,17,18,19,20]. Thus, OCP has been shown to activate multiple hard tissue–derived cells, whereas the effect of OCP on soft tissue–derived cells remain unknown. Regarding the effect of OCP on soft tissue–derived cells, the only evidence has been reported from in vivo implantation experiments using OCP dispersed in a gelatin sponge as a vehicle [21]. This study shows that OCP treatment–induced collagen and Sharpey fiber formation increase at the tendon-to-bone insertion, suggesting that OCP may stimulate tendon-related cells during enthesis tissue regeneration [21].

Tendon injuries are highly common and severe in the world [22], particularly the Achilles tendon, rotator cuff, and patellar tendon injuries, which have a high incidence because of sports or other strenuous physical activities [23]. Tendons are dense connective tissues that connect muscles and bones, transfer mechanical forces, and are essential for the integrity and function of the musculoskeletal system [24,25]. The structure of the tendon-to-bone attachment is known as the enthesis, which transfers muscle forces to the bone [26]. Enthesis is the weakest site in the early healing phase [27]. The surgical reattachment of the tendon to the bone frequently fails because of the lack of regeneration of the enthesis, which makes tendon-to-bone healing difficult. Tendon-to-bone reattaching and healing between two different types of tissues is a slow process compared to healing within homogenous tissue, such as bone healing [28]. The enhancement of the re-formation process of the attachment point is important to improve the healing of tendon-to-bone. Some studies suggest that enhanced tissue mineralization at the site of the tendon repair can contribute to tendon-to-bone healing [29]. Furthermore, formation of calcium phosphate deposition has been shown to be involved in enhancing tendon-to-bone healing [30,31,32]. However, tendon-bone ingrowth, mineralization, and maturation in the bone tunnel are not always satisfactory, which is another reason for poor tendon-bone healing [33,34,35].

Tendon stem/progenitor cells (TSPCs), a population of MSCs, is present in rat, mouse, rabbit, and human tendon tissues [36]. TSPCs have universal stem cell characteristics, such as colony-forming ability, self-renewal ability, and multi-differentiation potential, which enable them to differentiate into adipocytes, chondrocytes, and osteocytes [36,37,38]. The effect of TSPCs on tendon repair has been previously reported [39,40]. The potential advantage of TSPCs is that they are resident cells in tendons. Therefore, TSPCs as a cell source for tendon–bone junction healing is superior to other MSCs [41,42]. It has been demonstrated that the healing of the bone–tendon junction can be enhanced by adding Sharpey fibers using tendon stem cell sheets [43]. Bone tissue engineering can be a promising approach to repairing tendon–bone injuries. However, there is limited information on the application of bone substitute materials, such as calcium phosphate, for tendon-to-bone repair.

In this study, we culture rat TSPCs using two calcium phosphate materials. OCP and CDHA, obtained from the original OCP hydrolysis, with a lower Ca/P molar ratio compared to stoichiometric HA and similar particle morphology, are used to investigate the effect of calcium phosphate materials on TSPCs activation. Furthermore, TSPCs are cultured in calcium phosphate–conditioned media to exclude the effect of contact with OCP crystals on TSPCs. This study is designed to determine if OCP can stimulate soft tissue–derived TSPCs, as previously reported in multiple hard tissue–derived cells.

## 2. Results

### 2.1. Identification of Stemness in TSPCs

**Figure 1:**
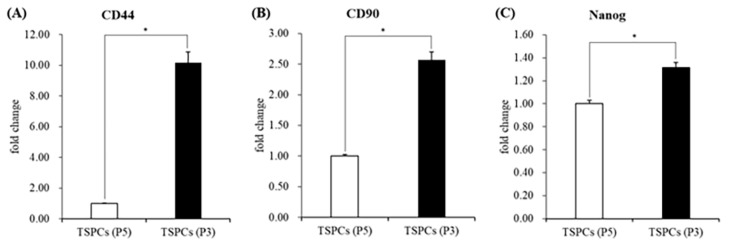
Identification of stemness in TSPCs by qPCR; (**A**–**C**) the expression of the mRNA of stemness-related marker genes (*CD44*, *CD90*, and *Nanog*). (*n* = 3), * *p* < 0.05.

The stemness of isolated rat TSPCs was assessed (Figure 1). The TSPCs at Passage 5 were used as a control group to test the expression of stem cell marker genes in TSPCs at Passage 3. The expression levels of the stem cell marker genes (*CD90*, *CD44*, and *Nanog*) were significantly higher at Passage 3 than at Passage 5.

### 2.2. Proliferation and Alkaline Phosphatase (ALP) Activities of TSPCs Treated with Calcium Phosphate Granules

**Figure 2:**
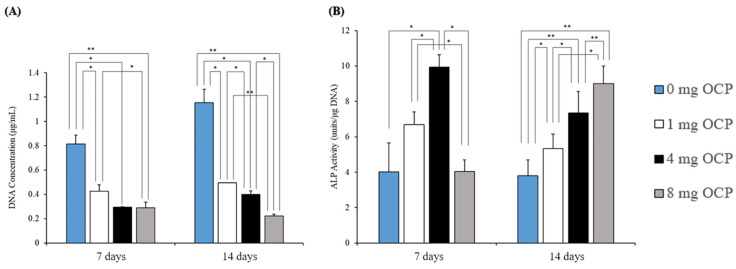
Responses of TSPCs to the different content of calcium phosphate granules; (**A**,**B**) the DNA content and ALP activity of TSPCs treated with different doses (0–8 mg) of OCP granules (*n* = 3) ** *p* < 0.01, * *p* < 0.05.

**Figure 3:**
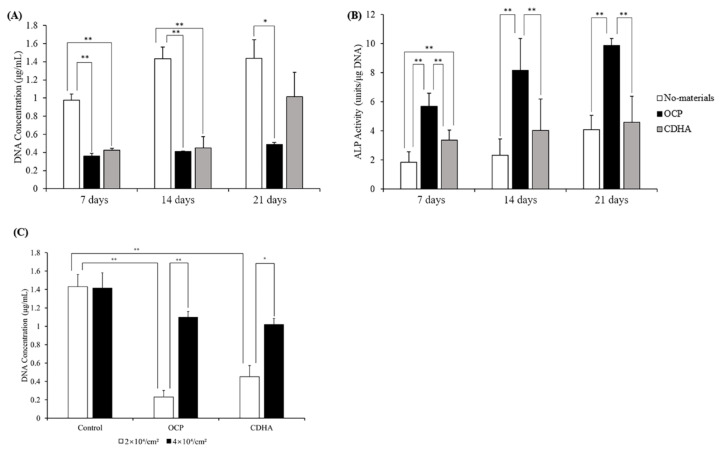
Responses of TSPCs to calcium phosphate granules; (**A**,**B**) the DNA (**A**) content and ALP activity (**B**) of TSPCs treated with or without 4 mg of OCP and CDHA granules; (**C**) the DNA content of different seeding density on day 14 (*n* = 3); ** *p* < 0.01, * *p* < 0.05.

TSPCs were confluent at a low seeding density (2 × 10^4^ cells/cm^2^) and cultured with 0, 1, 4, and 8 mg of OCP granules to evaluate the response of TSPCs according to the dose of OCP granules. As shown in Figure 2A, the deoxyribonucleic acid (DNA) content of TSPCs decreased with the increasing dose of the OCP granules (Figure 2A). The ALP activity in the 4 mg group was significantly higher than that in the other groups on day 7 (Figure 2B). However, the ALP activity increased with an increase in the dose of the OCP granules on day 14. Therefore, the treatment of TSPCs with 4 mg of OCP granules was considered the most appropriate dose for inducing osteoblast differentiation. Afterward, TSPCs were cultured with 4 mg of OCP or CDHA granules to investigate the effects of calcium phosphate granules on TSPCs at different time points (7, 14, and 21 days). As shown in Figure 3A, The DNA content in the OCP and CDHA groups was significantly lower than that in the control group on days 7 and 14. Although the DNA concentration increased in the CDHA group on day 21, it was constant in the OCP group over the incubation period. The ALP activity was significantly higher in the OCP group than in the other two groups (Figure 3B). The activity increased with the incubation period in both the OCP and control groups.

Additionally, the TSPCs were cultured with calcium phosphate granules at a high seeding density (4 × 10^4^ cells/cm^2^). The DNA content in the cells at the high seeding density significantly increased at a higher density than at a lower density (2 × 10^4^ cells/cm^2^) in the OCP and CDHA groups, and there was no significant difference with the control group (Figure 3C).

### 2.3. Osteogenic-Related Gene Expression and Calcified Nodules Formation of TSPCs Treated with Calcium Phosphate Granules

**Figure 4:**
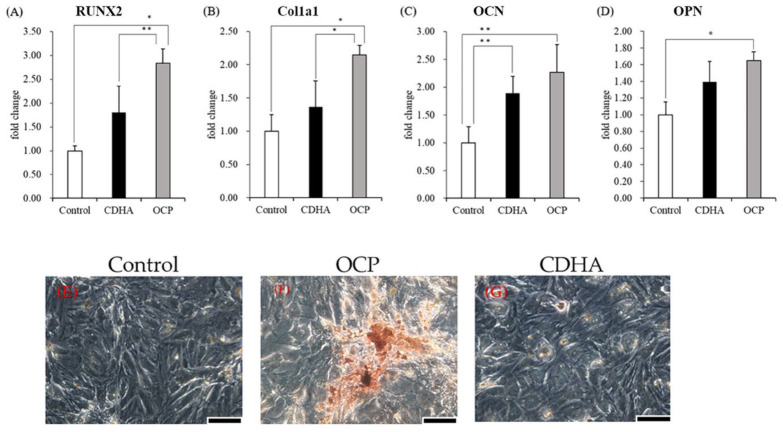
Effects of calcium phosphate granules for osteogenic differentiation in TSPCs; TSPCs treated with or without 4 mg of OCP and CDHA granules and cultured in cell culture insert system for 14 days; (**A**–**D**) the expression of the mRNA of osteogenic-related marker genes (*RUNX2*, *Col1a1*, *OCN*, *OPN*) (*n* = 3); TSPCs treated in the Control (**E**), OCP (**F**), and CDHA (**G**) group as visualized by calcium nodules (red) with Alizarin Red staining, scale bars = 20 μm, ** *p* < 0.01, * *p* < 0.05.

After incubating TSPCs treated with calcium phosphate granules for 14 days, the mRNA expression of osteoblastic differentiation markers was determined. The expression levels of *RUNX2* and *Col1a1* were significantly higher in the OCP group than in the control and CDHA groups (Figure 4A,B). The mRNA expression of *OCN* and *OPN* significantly increased in the OCP and CDHA groups than in the control group (Figure 4C,D). The expression levels of *OCN* and *OPN* were higher in the OCP group than in the CDHA group. However, the differences were not statistically significant. The incubated TSPCs stained with Alizarin Red were observed on day 14 (Figure 4E–G). Numerous calcified nodules formed in the OCP group compared to the control and CDHA groups.

### 2.4. Proliferation and ALP Activity of TSPCs Treated with Calcium Phosphate–Conditioned Media

**Figure 5:**
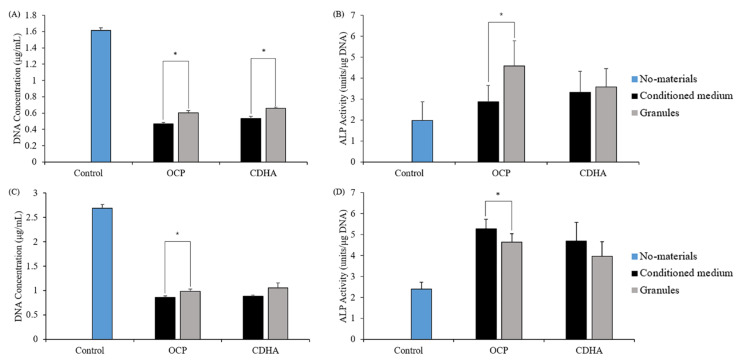
Responses of TSPCs to calcium phosphate conditioned medium; The DNA content of TSPCs treated with OCP or CDHA–conditioned medium for 7 days (**A**) and 14 days (**C**); the ALP activity of TSPCs treated with OCP or CDHA–conditioned medium for 7 days (**B**) and 14 days (**D**); (*n* = 3). *p* < 0.05.

TSPCs were treated in osteogenic or calcium phosphate–conditioned media for 7 and 14 days to investigate their responses to the chemical environment caused by the OCP and CDHA granules. Additionally, we compared the no-material, granule, and conditioned medium groups. The DNA content and ALP activity of the conditioned medium group were similar to those of the granule group (Figure 5). Furthermore, ALP activity in the OCP-conditioned medium group was higher than that in the OCP granules group on day 14 (Figure 5D).

### 2.5. Changes in Ion Compositions and the Degree of Supersaturation (DS) with Respect to the Calcium Phosphates in the Culture Media

The changes in the ion composition in the culture media were measured during the incubation of TSPCs (Table 1). The calcium ion (Ca^2+^) concentration in the media decreased in the OCP and CDHA groups after 3 days and slightly increased from days 9 to 15. The Ca^2+^ concentration was constant in the control group. The Ca^2+^ concentration was lower in the OCP group than in the CDHA group at the respective time points. The total concentration of inorganic phosphate (Pi) ions increased on day 3 in all groups. The concentration of Pi ions in the OCP and CDHA groups gradually decreased from days 9 to 15. The Pi ion concentration was higher in the OCP group than in the CDHA group, although the Pi ion concentration in the control group was the highest among all groups. The pH values of the media slightly increased after 3 days, and thereafter, there was no increase or decrease, although the ranges of pH change were small. However, the pH values were higher in the order of Control < OCP < CDHA at each incubation period.

We calculated the DS for HA, OCP, and dicalcium phosphate dihydrate (DCPD) in the supernatants of the cell culture media (Table 1). The DS values indicated that the supernatants were supersaturated with respect to OCP and HA in all groups before and after incubation. The DS with respect to HA was the highest (from 3.03 × 10^12^ to 8.39 × 10^13^), followed by the DS with respect to OCP (from 3.64 × 10^3^ to 6.25 × 10^4^) in all groups.

### 2.6. Characterization of the Calcium Phosphate Granules Incubated with the TSPCs

**Figure 6:**
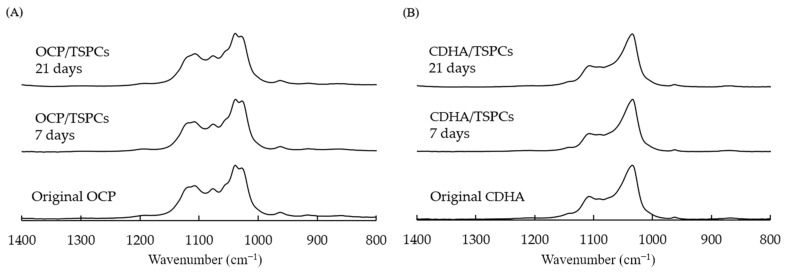
The change in the FTIR spectra of the OCP granules (**A**) or CDHA granules (**B**) before (original) and after immersion in TSPCs culture medium for 7 and 21 days.

The structures of the incubated OCP and CDHA were analyzed by Fourier transform infrared spectroscopy (FTIR). Figure 6 shows the FTIR spectra of OCP and CDHA before (original) and after the incubation with the TSPCs. The absorption bands attributed to ν_3_ PO_4_ at 1027 and 1038 cm^−1^, ν_3_ HPO_4_(5) at 1107, and ν_3_ HPO_4_(6) at 1117 cm^−1^ were detected in the spectrum of the original OCP (Figure 6A). The intensities of the bands attributed to ν_3_ PO_4_ at 1027 cm^−1^, ν_3_ HPO_4_(5), and ν_3_ HPO_4_(6) gradually decreased with the incubation period. The bands of ν_3_ PO_4_ and ν_3_ HPO_4_ were observed in the spectrum of the original CDHA at 1033 and 1106 cm^−1^, respectively (Figure 6B). However, the intensities of these bands were maintained in the CDHA spectra after the incubation.

## 3. Discussion

Currently, tendon repair–failure rates remain a difficult clinical challenge. Rotator cuff repair failure rates and anterior cruciate ligament (ACL) reconstruction failure rates have been reported to be 20% to 94% [44,45] and 10% to 25% [46,47], respectively. TSPCs have been suggested as seed cells for tendon tissue engineering [48]. However, no study has focused on the application of bone tissue engineering using OCP materials or determined if these materials affect the osteogenic differentiation of TSPCs to enhance tissue mineralization at the tendon repair enthesis and promote tendon–bone junction healing. In this study, we tested the hypothesis that OCP enhances the osteogenic differentiation of TSPCs in vitro, based on our previous in vivo studies [21]. We investigated the applicability of OCP as a new material to enhance tendon–bone junction healing, including the response of TSPCs to OCP, and the mechanism by which the chemical environment induced by OCP may promote the osteogenic differentiation of TSPCs in vitro was investigated.

TSPCs have been demonstrated to have osteogenic differentiation potential [38]. However, it has been reported that *CD90* surface expression is downregulated during the in vitro passaging of rat patellar TSPCs. Therefore, experiments with early and consistent cell passaging are recommended for reproducible results [49]. To ensure that the TSPCs at passage 3 used in all experiments could be induced to differentiate, we identified the stemness of cells and compared it with that of TSPCs at passage 5. The quantitative polymerase chain reaction (qPCR) results showed that the TSPCs at passage 3 had a relatively high expression of cell stemness marker genes (Figure 1). In general, TSPCs seeded at relatively high densities show a high fold–increase in cell number than cells seeded at relatively low densities [50,51]. Interestingly, under the influence of OCP granules, the proliferation of TSPCs seeded at low density was inhibited 6–7 days before and returned to normal. The inhibition of cell proliferation increased with an increase in the amount of OCP granules (Figure 2). However, the proliferation of TSPCs seeded at high densities was minimally affected, and there was little difference in cell numbers compared with the control group on day 14 (Figure 3). This indicated that OCP has a minimal effect on the proliferation of TSPCs in general. However, when the number of TSPCs is small, OCP might inhibit the early proliferation of TSPCs. After treatment and culture with OCP granules, the expression of the ALP activity, the mRNA expression of osteogenic-related marker genes (*RUNX2, Col1a1, OCN, OPN*), and the mineralized nodules of TSPCs increased (Figure 4). Additionally, the expression of ALP activity was higher with an increase in the dose of OCP granules, which indicated that OCP promoted the osteogenic differentiation of TSPCs in a dose-dependent manner (Figure 2). Our previous study showed that OCP treatment–induced Sharpey fibers increased at the tendon-to-bone insertion [21], which is regarded as the earliest sign of osseointegration [28,52]. The present results demonstrated the low toxicity of OCP to TSPCs and the ability to promote the osteogenic differentiation of TSPCs.

OCP materials have osteoconductive and biodegradable properties, can release Pi ions, and absorb calcium ions stably during their conversion into CDHA through a hydrolysis reaction in neutral aqueous conditions [8,11,17]. To elucidate the mechanism by which OCP promotes the osteogenic differentiation of TSPCs, we evaluated changes in the pH, ionic concentration, and crystal structure of calcium phosphate in the culture medium before and after calcium phosphate addition, which allowed us to determine the DS values to express the correlation between OCP hydrolysis and solution (Figure 6, Table 1). The DS with respect to HA at days 3, 9, and 15 of the TSPCs culture was higher in the OCP group than in the CDHA group, suggesting that the OCP granules in the cultured medium are in a microenvironment that promotes HA precipitation and the conversion of OCP to HA. FTIR analysis showed that OCP tends to convert to CDHA during TSPCs culture (Figure 6). With reference to our previous study [53], we cultured TSPCs in OCP-soaked conditioned medium and observed the effect of OCP on the osteogenic differentiation of TSPCs (Figure 5). The results suggested that the microenvironment of OCP crystal hydrolysis may contribute to the osteogenic differentiation of TSPCs.

To create a persistent and appropriate microenvironment to regulate differentiation in vivo, the selection of cell/molecular delivery vehicles or biomaterial scaffolds is important to repair tendon injuries. Such materials should have low toxicity, compatibility with native tissues, and biodegradability over time as regeneration or repair proceeds, which could be promising approaches for tendon-repair therapy [54]. Our results suggest that OCP has desirable characteristics for the repair of tendon injuries. Although we attempted to use OCP to promote the osteogenic differentiation of TSPCs to accelerate the repair of tendon–bone injury, the tendon–bone structure is very complex, and it is still very limiting to study the tendon–bone interface repair only from the direction in which the chemical environment affects the osteogenic differentiation potential of TSPCs. It has been reported that the potential of TSPCs to differentiate into fibrocartilage-like cells may contribute to the promotion of tendon injury repair [55]. Studies on OCP repair tendon–bone injury may be focused on OCP, which enhances the fibrocartilage and chondrogenic differentiation potential of TSPCs. Additionally, we only focused on the effect of the chemical environment of OCP hydrolysis on TSPCs and did not study the effect of OCP on the osteogenic differentiation of TSPCs by affecting protein signaling pathways. Studies have shown that Wnt/𝛽-catenin plays an important role in bone development, and therefore, it has received widespread attention [56,57,58]. Some studies have shown the feasibility of this research direction [59].

In conclusion, in this study, the microenvironment of OCP hydrolysis can enhance the osteogenic differentiation of TSPCs, which can be applied to treat TSPC-based bone–tendon junction repair if OCP materials are utilized.

## 4. Materials and Methods

### 4.1. Synthesis of Calcium Phosphate Granules

OCP and CDHA were synthesized by a wet synthesis method, as previously described [6,60]. CDHA was obtained through hydrolysis in hot water. Briefly, OCP slurry was continuously mixed in a water bath at 65 °C for 48 h. The obtained OCP and CDHA were sieved between 32 and 48 mesh to prepare calcium phosphate granules with sizes of 300–500 μm. The granules were sterilized using a dry sterilizer at 120 °C for 2 h.

### 4.2. TSPCs Isolation, Identification, and Culture

Eight-week-old healthy male Sprague–Dawley rats were used to isolate TSPCs. All animal experimental protocols were approved by the Animal Research Committee of Tohoku University (approval number: 2020DnA-009). The TSPCs were isolated according to previously established procedures [36]. Briefly, the midsubstance of Achilles tendon tissues was collected. The collected tissues were minced and digested for 2.5 h at 37 °C with 3 mg/mL of type I collagenase (Sigma-Aldrich, St. Louis, MO, USA). Afterward, the cells were passed through a cell strainer (70 μm pore size, Becton, Franklin Lakes, NJ, USA) to obtain a single-cell suspension. The isolated cells were washed and centrifuged to resuspend in low-glucose Dulbecco’s Modified Eagle’s medium (FUJIFILM Wako Pure Chemical Co., Osaka, Japan) containing 10% fetal bovine serum (FBS; Gibco, Waltham, MA, USA) and 1% penicillin/streptomycin solution (Nacalai Tesque Inc., Kyoto, Japan) as the complete medium at 37 °C in a 5% CO_2_ incubator. The cells at passages 3 and 5 (P3, P5) were used for all subsequent experiments. P5 cells served as a control group, and the stemness of TSPCs was identified by measuring the mRNA expression of *CD44*, *CD90*, and *Nanog* using a qPCR. The culture medium was replaced every 3 days during the experiments.

### 4.3. Analysis of the Proliferation and Osteoblastic Differentiation of TSPCs Cultured with Calcium Phosphate Granules

To investigate the presence or the pre-presence of OCP and CDHA crystals in the media on TSPCs activities, the cultures in the presence and the pre-presence of these crystals were conducted as explained in this section and the next Section 4.4, respectively. TSPCs (P3) were seeded at 2 × 10^4^ cells/cm^2^ in 24-well plates and cultured in the complete medium. After cell confluence, the cells were incubated with 0, 1, 4, and 8 mg of OCP or 4 mg of CDHA using a Transwell system with 3.0 μm pore size (FALCON^®^ Cell Culture Insert, Corning Inc., Corning, NY, USA) in 1 mL of osteogenic medium in 5% CO_2_ and 95% air atmosphere under humidified conditions at 37 °C for 7, 14, and 21 days. The osteogenic medium was the complete basal medium supplemented with 100 nM of dexamethasone (Sigma-Aldrich, St. Louis, MO, USA), 50 μM of L-ascorbic acid 2-phosphate sesquimagnesium salt hydrate (Sigma-Aldrich, St. Louis, MO, USA), and 10 mM of disodium β-glycerophosphate tetrahydrate (Tokyo Chemical Industry, Tokyo, Japan). The cell culture medium was changed every 3 days. After the cultivation, the DNA concentrations and ALP activities of the cells were determined. In order to compare the cell proliferation at different seeding densities, the cells were also incubated in 1 mL of osteogenic media in the presence of with 4 mg of OCP and CDHA after the confluence of cells seeding at 4 × 10^4^ cells/cm^2^ in the complete media.

The TSPCs at passage 3 were seeded at a density of 4 × 10^4^ cells/cm^2^ in 24-well plates and cultured in the complete medium until confluence. Subsequently, they were cultured with 4 mg of OCP or CDHA granules in 1 mL of osteogenic medium for 14 days to assess the mRNA expression of osteoblastic differentiation markers (*RUNX2*, *Col1a1*, *OCN*, and *OPN*) by qPCR. The incubated cells were stained with Alizarin Red S to observe the calcium nodule formation.

### 4.4. Analysis of the Osteoblastic Differentiation of TSPCs Treated with Conditioned Media

The dose of calcium phosphate granules was adjusted to 4 mg, and the granules were soaked in 1 mL of complete basal medium in each well of a 24-well plate for 24 h at 37 °C. The calcium phosphate–conditioned medium was prepared by mixing of 500 μL of the medium incubated with granules and 500 μL of osteogenic medium. TSPCs were seeded in 24-well plates and cultured in the calcium phosphate-conditioned media in 5% CO_2_ and 95% air atmosphere under humidified conditions at 37 °C for 7 and 14 days. The DNA concentration and ALP activity of the cells were measured for each incubation period.

### 4.5. DNA Concentration Measurement and ALP Activity Assay

Osteoblastic differentiation of TSPCs was estimated by measuring ALP activity per living cell to standardize the effect of OCP and CDHA on osteogenic differentiation from TSPCs. The incubated TSPCs were lysed in 250 μL of 0.2% Triton X-100 solution (Sigma-Aldrich, St. Louis, MO, USA) and sonicated. The proliferation of TSPCs was tested by quantifying the DNA concentration using Quant-iT^TM^ PicoGreen^®^ dsDNA Reagent and Kits (Invitrogen, Waltham, MA, USA) according to the manufacturer’s instructions. The ALP activity in the incubated cells was determined using LabAssay ALP^®^ (FUJIFILM Wako Pure Chemical Co., Osaka, Japan) and calculated according to the manufacturer’s instructions.

### 4.6. Real-Time qPCR

Total RNA was isolated from the incubated cells using TRIzol Reagent (Thermo Fisher Scientific, Waltham, MA, USA) according to the manufacturer’s instructions. RNA was reverse-transcribed into cDNA using a ReverTra Ace^®^ qPCR RT Master Mix with a gDNA remover (TOYOBO Life Science, Osaka, Japan). Optimal oligonucleotide primers and TaqMan probes were designed using the ProbeFinder software (Roche Diagnostics, Basel, Switzerland). The sequences of the PCR primers and universal probes are listed in Table 2. Real-time TaqMan PCR (RT-PCR) was used to evaluate stemness-related (*CD44*, *CD90*, and *Nanog*) and osteogenic-related marker (*RUNX2*, *Col1a1*, *OCN*, and *OPN*) gene expressions in TSPCs. The RT-PCR was conducted in a 20 μL reaction volume with 1× FastStart Essential DNA Probe Master (Roche, Basel, Switzerland), 500 nM each of forward and reverse primers, 200 nM Universal ProbeLibrary probe, and the cDNA template. Cycling conditions were as follows: 45 cycles of 95 °C for 10 s, 60 °C for 30 s, and 72 °C for 1 s. The RT-PCR was performed using LightCycler 1.5 (Roche Applied Science, Mannheim, Germany). The expression of every target gene was normalized to that of *GAPDH*.

### 4.7. Alizarin Red Staining

The culture medium was removed, and the cells were washed twice with phosphate-buffered saline (PBS). The cells were fixed in 75% ethanol solution at −20 °C for 1 h and then stained with Alizarin Red (Sigma-Aldrich, St. Louis, MO, USA) according to the instructions. The images of the stained cells were captured using an optical microscope (Leica DMI 4000 B, Leica Microsystems GmbH, Wetzlar, Germany).

### 4.8. FTIR Analysis of the Calcium Phosphate Granules Incubated with TSPCs

The granules of OCP and CDHA incubated with the cells were collected on days 7 and 21. The granules were washed with pure water and lyophilized. The sample was diluted in potassium bromide and analyzed using an FTIR spectroscopy (FT/IR-4600; JASCO Corporation, Tokyo, Japan) over a range of 4000–400 cm^−1^ at a 4 cm^−1^ resolution.

### 4.9. Measurement of Ion Composition and the Calculation of the DS with Respect to Calcium Phosphates in the Cell Culture Environment

The supernatants of the culture medium were collected on days 3, 9, and 15. The Ca^2+^ and Pi ion concentrations in the supernatants were determined using Calcium E and Phosphor C tests (FUJIFILM Wako Pure Chemical Co.), respectively. The pH of the supernatant was measured using a pH electrode (9618S-10D; HORIBA, Ltd., Kyoto, Japan).

The solubilities of OCP and CDHA in the culture media of TSPCs were estimated by calculating the DS with respect to OCP, HA, and DCPD phases in the collected supernatants. The DS can be calculated by dividing the ionic product by the solubility product constant with respect to the calcium phosphate phases. According to previous reports [61,62,63], the DS was calculated from the analysis results for [Ca], [Mg], [Na], [K], [Pi], [Cl], and [F], and the pH value based on the three mass balance equations for [Ca], [P], and [Mg]. In this study, the pH and [Ca] and [Pi] were used to calculate the DS. The calculations indicated the presence of HCO_3_^−^ in the medium. The presence of CaH_2_PO_4_^+^, CaHPO_4_^0^, MgHPO_4_^0^, CaHCO_3_^+^, and MgHCO_3_^+^ was considered in the supernatants. The DS was estimated from the mean ionic activity products with respect to OCP, HA, and DCPD. Assuming that the Na^+^ concentration was the background electrolyte, the ionic strength was set at 150 mM. The values of [Mg] and [F] were assumed to be approximately zero. The solubility product constants used were 7.36  ×  10^−60^ (mol/L)^9^ for HA [64], 2.51  ×  10^−49^ (mol/L)^8^ for OCP [65], and 2.77 × 10^−7^ (mol/L)^2^ for DCPD [66] at 37 °C.

### 4.10. Statistical Analysis

The results are shown as mean ± standard deviation (SD). Tukey–Kramer multiple comparison analysis was performed to analyze the statistical differences among multiple groups. Furthermore, the student’s t-test was used to evaluate the statistical differences between the two groups. For all comparisons, *p*-values less than 0.05 were considered statistically significant.

## 5. Conclusions

In this study, we demonstrated that OCP can promote the osteogenic differentiation of TSPCs. The microenvironment affected by OCP significantly promoted the osteogenic differentiation of TSPCs, which seems to be related to the changes in the ionic concentration and pH in the chemical environment caused during the hydrolysis of OCP.

## Figures and Tables

**Table 1:** Solution composition (Ca^2+^, Pi ion, and pH) and degree of supersaturation (DS) with respect to calcium phosphates in culture media incubated with OCP and CDHA.

	Period (Days)	Ca^2+^ (mM)	Pi (mM)	pH	DS at pH (Each) and 37 °C		
					HA	OCP	DCPD
DMEM	0	1.85	1.12	7.59	4.16 × 10^12^	5.24 × 10^3^	6.83 × 10^−1^
Control	3	1.93	1.88	7.65	3.87 × 10^13^	3.64 × 10^3^	1.18 × 10^0^
OCP	3	1.20	1.65	7.66	3.03 × 10^12^	4.34 × 10^3^	6.69 × 10^−1^
CDHA	3	1.50	1.47	7.68	7.94 × 10^12^	8.24 × 10^3^	7.44 × 10^−1^
Control	9	2.10	1.92	7.62	4.55 × 10^13^	4.52 × 10^4^	1.29 × 10^0^
OCP	9	1.43	1.59	7.63	4.76 × 10^12^	6.55 × 10^3^	7.57 × 10^−1^
CDHA	9	1.61	1.44	7.65	7.84 × 10^12^	8.69 × 10^3^	7.75 × 10^−1^
Control	15	2.12	1.90	7.68	8.39 × 10^13^	6.25 × 10^4^	1.31 × 10^0^
OCP	15	1.52	1.54	7.69	1.06 × 10^13^	1.04 × 10^4^	7.90 × 10^−1^
CDHA	15	1.63	1.40	7.70	1.26 × 10^13^	1.10 × 10^4^	7.72 × 10^−1^

**Table 2:** Primer sequences and conditions for Taqman real-time PCR analysis in this study.

Gene	Primer Sequences	Universal Probe
*CD44*	F 5′-GGATGACGCCTTCTTTATTGG-3′ R 5′-TGTTGCATGGCTTTTTGAGT-3′	#49
*CD90*	F 5′-CCACAAGCTCCAATAAAACTATCAA-3′ R 5′-AGCAGCCAGGAAGTGTTTTG-3′	#40
*Nanog*	F 5′-TGCACTCAAGGATAGGTTTCAG-3′ R 5′-TTTGGAACCAGGTCTTCACC-3′	#67
*Col1a1*	F 5′-TCCTGGCAAGAACGGAGAT-3′ R 5′-CAGGAGGTCCACGCTCAC-3′	#60
*OCN*	F 5′-ATAGACTCCGGCGCTACCTC-3′ R 5′-CCAGGGGATCTGGGTAGG-3′	#125
*OPN*	F 5′-GAGTTTGGCAGCTCAGAGGA-3′ R 5′-TCTGCTTCTGAGATGGGTCA-3′	#41
*RUNX2*	F 5′-CCACAGAGCTATTAAAGTGACAGTG-3′ R 5′-AACAAACTAGGTTTAGAGTCATCAAGC-3′	#98
*GAPDH*	F 5′-TGGGAAGCTGGTCATCAAC-3′ R 5′-GCATCACCCCATTTGATGTT-3′	#9

## Data Availability

The data were provided in the manuscript.
